# Identification and Expression Analysis of Polyphenol Oxidase Gene Family Members in Response to Wound Stress in Lettuce (*Lactuca sativa* L.)

**DOI:** 10.3390/plants14060972

**Published:** 2025-03-19

**Authors:** Mei Guo, Yueming Tang, Yiwen Yang, Jinghong Luo, Jia Gao

**Affiliations:** 1 College of Food and Bioengineering, Chengdu University, Chengdu 610106, China; meiguo9806@163.com; 2 Institute of Agro-Products Processing Science and Technology, Institute of Food Nutrition and Health, Sichuan Academy of Agricultural Sciences, Chengdu 610066, China; ymtang912@163.com (Y.T.); yiweny1024@163.com (Y.Y.); mirror28@163.com (J.L.); 3 Sichuan Advanced Agricultural & Industrial Institute, China Agricultural University, Chengdu 611430, China; 4 Sichuan Research Center of Vegetable Engineering and Technology, Chengdu 611934, China

**Keywords:** lettuce, browning, polyphenol oxidase, expression pattern

## Abstract

Mechanical injury to lettuce often leads to enzymatic browning caused by polyphenol oxidase (PPO), significantly impairing its sensory quality and processing suitability. In this study, the *LsPPOs* gene family was comprehensively identified and characterized using bioinformatics methods, including gene and protein structure, codon usage bias, phylogenetic relationships, and gene expression in response to wound stress. Further analysis of the relationship between *LsPPOs* expression profile and browning was performed. A total of 17 *LsPPO* family members (*LsPPO1-LsPPO17*) were identified from publicly available lettuce databases, encoding proteins ranging from 146 to 667 amino acids, with a G/C bias. Most were localized in the chloroplast. The motif structure was highly conserved among family members, and phylogenetic analysis revealed four distinct groups. All genes lacked introns, except *LsPPO2* which contained an intron. After mechanical injury, browning at the stem site deepened over time, with PPO activity increasing. The majority of *PPO* members were significantly upregulated after fresh-cut processing. Among them, *LsPPO3*, *LsPPO4*, and *LsPPO12* showed sustained upregulation, exhibiting a strong positive correlation with the browning phenotype and PPO activity. Notably, *LsPPO4* demonstrated the highest transcriptional abundance and upregulation in response to a wound, indicating its major role in lettuce stem browning. The results of this study provide a foundation for further investigation into the functional role of *LsPPOs* and support the development of lettuce varieties with enhanced resistance to browning.

## 1. Introduction

Lettuce (*Lactuca sativa* L.), an annual or biennial vegetable crop belonging to the Asteraceae family, is rich in various nutrients such as vitamins, minerals, and dietary fiber. Over the past five years, its global production has remained stable at approximately 27 million tons annually, with China contributing roughly 50% of the world’s output (https://www.fao.org/faostat/ accessed on 16 February 2025) [1,2]. Lettuce is commonly consumed raw due to its slightly sweet, crisp, and refreshing texture, often featured in vegetable salads. However, fresh-cut produce has a limited shelf life, and processing can cause varying degrees of damage, including browning at cut surfaces, moisture loss, and microbial contamination. Mechanical injury to lettuce disrupts cellular structure, accelerating physiological metabolism and oxidative processes. The secretion of phenolic compounds at cut sites reacts with oxidative enzymes, leading to browning, which significantly impacts consumer perception and reduces market value [3,4]. Within the lettuce supply chain, product quality deteriorates within just three days of retail [5]. Thus, maintaining the visual appeal of lettuce is a pressing challenge.

Polyphenol oxidase (PPO), a copper-containing enzyme encoded by nuclear genes, is widely distributed in nature and found in plants, animals, and fungi [6,7]. In plants, PPO is encoded by multiple nuclear genes, often exhibiting family characteristics, with *PPO* families of varying sizes present in terrestrial plants except for the *Arabidopsis* genus [8]. PPO plays diverse physiological roles, including pest resistance, flower pigmentation, wound healing, and abiotic stress tolerance [9,10,11]. Additionally, PPO is a key enzyme responsible for enzymatic browning in fruits and vegetables. It oxidizes phenolic compounds into o-diphenols and subsequently into o-quinones, which polymerize to form melanin [12]. In cucurbits, several *PPO* genes have been characterized, revealing that all of them are involved in pigmentation and stress responses. For instance, in luffa (*Luffa cylindrical*) multiple *LcPPO* genes were identified. All of them play a crucial role in browning and other functions in luffa [13]. Similarly, in melons (*Cucumis melo* L.) the presence of *PPO* homologs has been highlighted in relation to browning regulation and pigment synthesis [14]. Beyond cucurbits, homologous *PPO* genes have been studied in other crops. For example, in apples, potatoes, and lotus roots, wounding stress also activated *PPO* expression, significantly increasing its activity and resulting in reddish-brown discoloration at cut surfaces [15,16,17].

Targeted inactivation of PPO through genetic engineering to reduce phenolic oxidation represents an effective anti-browning strategy. Studies have demonstrated that regulating *PPO* genes in plants using RNA interference (RNAi) and gene editing technologies can significantly lower its activity. For instance, introducing vectors containing *PPO* gene interference fragments into apple plants has successfully produced browning-resistant materials [18]. Similarly, CRISPR/Cas9-mediated knockout of *PPO* genes in potatoes, eggplants, and mushrooms has reduced PPO activity and browning in cut tissues [19,20,21].

Bioinformatics plays a key role in gene family analysis. It can systematically reveal the structure, function, and evolutionary relationships of gene families by integrating genomics, transcriptomics, and proteomics data in multiple dimensions. This not only helps to understand the characteristics of genes across species, but also provides a molecular basis for unraveling complex biology [22]. For example, by comparing the gene family members of different species, it can be inferred that the functional differentiation and duplication events of genes are conducive to the preservation and cultivation of good species [23].

Therefore, *PPO* knockout based on gene editing is a promising strategy to mitigate stem browning and preserve lettuce quality. To this end, identifying *PPO* gene family members in lettuce and understanding their expression patterns are crucial. This study employs bioinformatics to characterize the lettuce *PPO* family, analyzing sequence features, phylogenetic relationships, and expression patterns under wound stress. By exploring the relationship between *PPO* family members and enzymatic activity, we aim to elucidate the role of *LsPPOs* in lettuce stem browning, providing a theoretical foundation for developing browning-resistant lettuce cultivars.

## 2. Results

### 2.1. Identification and Characteristics of PPO Genes in Lettuce

A total of 17 genes containing PPO specific conserved domains—tyrosinase (PF00264), PPO1_DWL (PF12142), and PPO1_KFDV (PF12143)—were obtained through a search, and named *LsPPO1-17*. In order to clearly understand the characteristics of *LsPPOs* family in lettuce, we analyzed gene length, coding sequence length (CDS), amino acid, molecular weight, average value of total hydrophilicity, isoelectric point, instability coefficient, chromosome localization, subcellular localization (Table 1), and protein secondary structure prediction (Table S1) of these genes encoding proteins.

The coding sequences ranged from 622 to 2137 bp in length, encoding proteins ranging from 146 to 667 amino acids, with molecular weights between 38.42 and 43.07 KDa. Among these, *LsPPO14* had the longest sequence and encoded the highest number of amino acids, while *LsPPO3* had the shortest sequence and encoded the fewest amino acids. The average hydrophilicity of all *LsPPOs* was negative, indicating that they were hydrophilic proteins. Their theoretical isoelectric points ranged from 4.73 to 8.32, with an average of 6.3, suggesting that most *LsPPOs* were acidic proteins. The instability indices varied from 32.42 to 51.66, with *LsPPO14* being the most stable and *LsPPO3* the least stable.

The distribution of *LsPPOs* across chromosomes was uneven. Members of the *LsPPOs* family were located on chromosomes two, three, four, seven, eight, and nine, with the highest density observed on chromosomes four, seven, and nine. Specifically, *LsPPO3* to *LsPPO6* were found on chromosome four, *LsPPO7* to *LsPPO9* on chromosome seven, and *LsPPO11* to *LsPPO17* on chromosome nine. In contrast, *LsPPO1*, *LsPPO2*, and *LsPPO10* were located on chromosomes two, three, and eight, respectively. Subcellular localization predictions revealed that LsPPOs proteins were primarily localized in the chloroplast (Chlo), with some also found in other organelles such as the cytoskeleton, mitochondria, and peroxisomes.

As shown in Table S1, the proteins encoded by the LsPPOs consisted of four structural elements: α-helices, β-turns, extended chains, and random coils. The proportion of α-helices ranged from 14.93% to 19.86%, β-turns from 0.32% to 2.74%, extended chains from 11.34% to 23.97%, and random coils from 52.42% to 72.99%. The β-folds might include key sites for substrate interaction during catalysis [24].

### 2.2. Evolutionary Analysis of LsPPOs Gene Family in Lettuce

**Figure 1:**
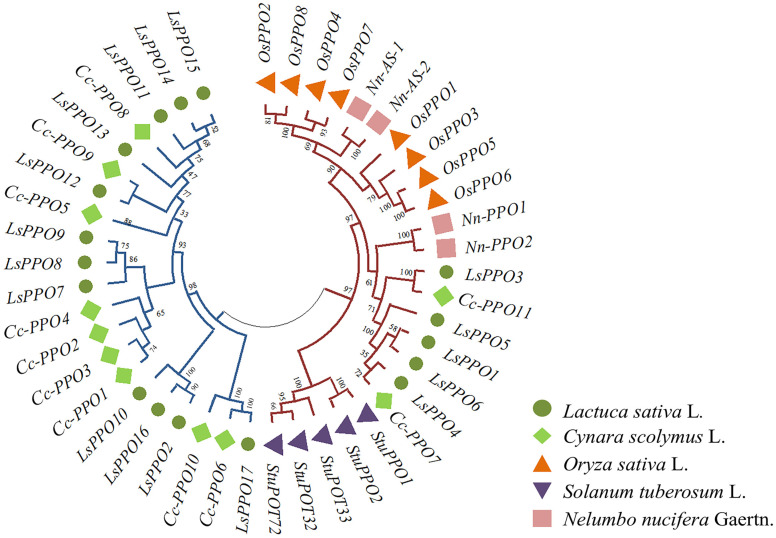
Phylogenetic tree between lettuce LsPPOs family and other species; the red and blue lines on phylogenetic tree represent A subgroup and B subgroup, respectively.

To further investigate the evolutionary relationships among the LsPPOs in lettuce, the PPO amino acid sequences of artichoke (*Cynara cardunculis* L., 11 CcPPOs), rice (*Oryza sativa* L., 8 OsPPOs), potato (*Solanum tuberosum* L., 5 StuPPOs), and lotus (*Nelumbo nucifera* Gaertn, 4 NnPPOs) were analyzed (Figure 1). These PPOs were classified into two subgroups (A and B). Subgroup A consisted exclusively of lettuce and artichoke PPOs, while subgroup B grouped together PPOs from potato, rice, lotus, as well as two artichoke CcPPO members and five lettuce LsPPO members. Notably, artichoke consistently clustered with lettuce, suggesting a closer evolutionary relationship between these two species. Additionally, LsPPO1, LsPPO3, LsPPO4, LsPPO5, and LsPPO6 separated from the 12 other lettuce PPOs and clustered with PPOs from other species. This separation implied functional divergence within the PPO family. These five genes from lettuce potentially shared similar functions with PPOs from artichoke, potato, and lotus.

### 2.3. Predictive Analysis of Gene Structure and Protein Conserved Motifs of Lettuce LsPPOs Family

**Figure 2:**
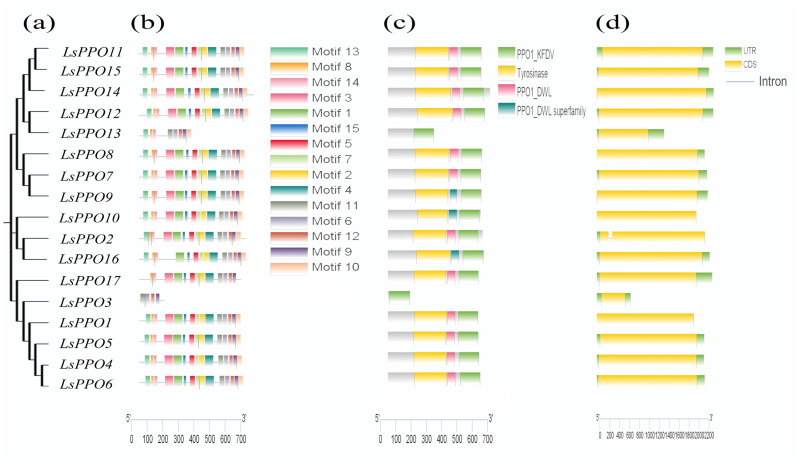
Phylogeny (**a**) and conserved motif analysis (**b**) of *LsPPOs*; conserved domain analysis (**c**); for gene structure analysis (**d**), the green box represents the UTR, the yellow box represents the exon, and the - represents the intron.

To examine the structural features of the LsPPO gene family members, we analyzed their domains, conserved motifs, and secondary structures (Figure 2). Motif analysis revealed that all family members shared four conserved motifs (motif6, motif9, motif11, and motif12) (Figure 2b), indicating that these motifs were relatively conserved throughout the evolutionary history of the PPO gene family. The C-terminal region of LsPPO proteins was highly conserved, corresponding to the PPO1_KFDV (PF12143.11) domain. In contrast, motifs13, 3, and 10 were missing in some genes, suggesting their potential functional differences.

CDD analysis from NCBI showed that all proteins contained the PPO1_KFDV (PF12143.11) domain. With the exception of LsPPO3 and LsPPO13, most proteins also harbored the tyrosinase (PF00264.23) and PPO1_DWL (PF12142.11) domains. Secondary structure predictions provided further insights into the functional aspects of these proteins.

As shown in Figure 2d, the gene structure analysis revealed that most *LsPPO* genes shared a similar structure. With the exception of *LsPPO1* and *LsPPO10*, all other *LsPPO* genes had untranslated regions (UTRs) at both the 5′ and 3′ ends. Additionally, while *LsPPO2* contained one intron, the remaining genes lacked introns. These findings suggest that the *LsPPO* genes in lettuce are generally conserved, with some exhibiting structural diversity.

### 2.4. Analysis of PPO Gene Codon Bias in Lettuce

Codon usage bias analysis of the *LsPPO* genes in lettuce was conducted online, with the results summarized in Table S2. The average GC content of the *LsPPO* genes ranged from 39.61% to 49.44%, with *LsPPO3* and *LsPPO17* exhibiting the lowest and highest GC, respectively. The GC content at the first, second, and third codon positions (GC1, GC2, and GC3) varied from 30.93% to 51.93%, 36.18% to 41.55%, and 39.09% to 49.44%, respectively, with the average order of GC1 > GC3 > GC2. The effective number of codons (ENC) for all genes was greater than the threshold value of 35, ranging from a minimum of 39.61 (*LsPPO3*) to a maximum of 60.31 (*LsPPO10*).

As shown in Table S3, a total of 61 synonymous codons were identified in the 17 *LsPPO* genes (excluding methionine codons CUG and UUG, as well as the three stop codons UAA, UAG, and UGA). Among these, 31 codons had an RSCU (relative synonymous codon usage) value greater than one, indicating higher frequency usage. The RSCU values for UGA, UUG, AUG, AGA, and AGG were all greater than 1.5, reflecting a strong preference for these codons. Notably, the RSCU value for AUG was the highest at three, indicating the strongest preference for this codon.

### 2.5. Changes in Lettuce Color and PPO Activity

**Figure 3:**
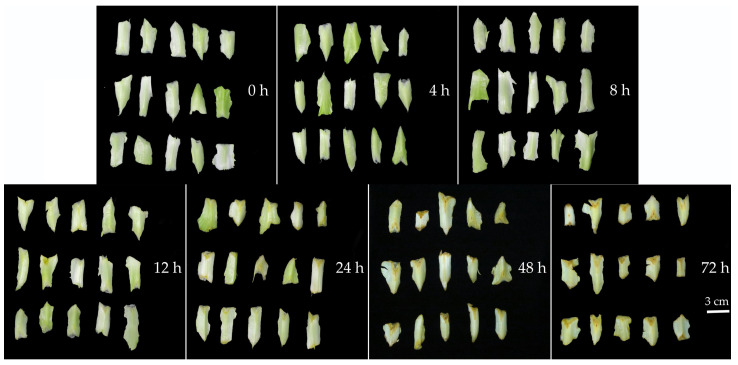
Color change in lettuce stem (3~5 cm) stored at room temperature (25 ± 1 °C, relative humidity 86% ± 3%, dark storage) for 72 h. h represents hour.

**Figure 4:**
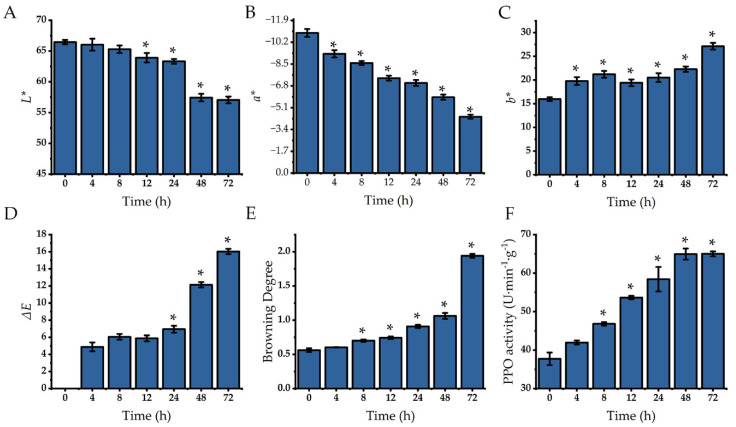
Changes in color parameters and PPO activity of fresh-cut lettuce stems during 72 h storage at room temperature (25 ± 1 °C, relative humidity 86% ± 3%, dark storage). L*, Luminosity (**A**); a*, red/green (**B**); b*, yellow/blue (**C**); ∆*E*, for differences in color empfindung (**D**); browning degree (**E**); PPO activity (**F**). * represents that there are significant differences between different time points and 0 h (*p* < 0.05).

As shown in Figure 3, under room temperature (25 ± 1 °C, relative humidity 86% ± 3%, dark storage) storage conditions, slight browning began to appear at the cut edges of the lettuce after 12 h of storage. The degree of browning gradually deepened, becoming more pronounced after 48 h of storage. Color difference measurements of the lettuce cut surface (Figure 4A–C) showed that as storage time increased, the L* value gradually decreased, while the a* and b* values increased. Based on the changes in L*, a*, and b* values, the ∆*E* value also progressively increased (Figure 4D). Further assessment revealed that the degree of browning increased with longer storage time (Figure 4E). The color difference and browning degree results were consistent with the visual observations (Figure 3).

A gradual increase was observed in PPO activity during the 72 h storage of lettuce at room temperature (Figure 4F). At 72 h, the activity was 1.72 times that at 0 h.

### 2.6. Expression Analysis of Lettuce LsPPOs

**Figure 5:**
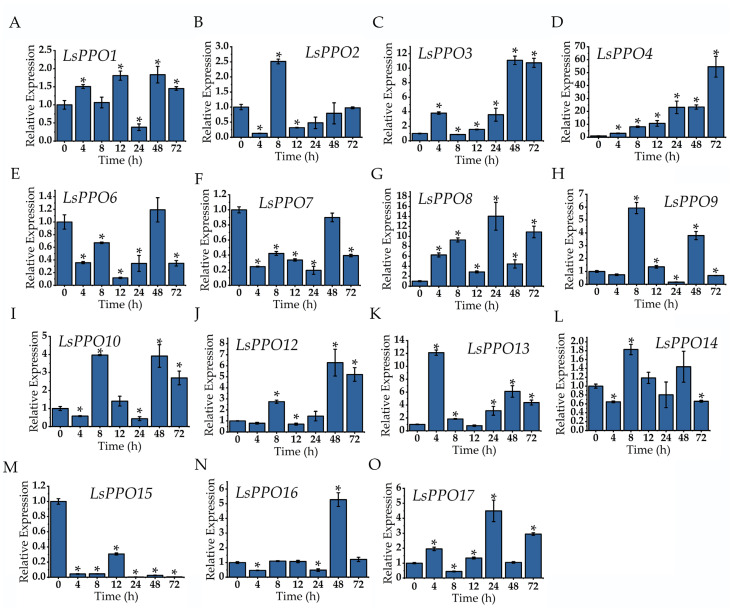
Relative expression of *LsPPOs* in lettuce stems stored at room temperature (25 ± 1 °C, relative humidity 86% ± 3%, dark storage) for 72 h. *LsPPO1* (**A**); *LsPPO2* (**B**); *LsPPO3* (**C**); *LsPPO4* (**D**); *LsPPO6* (**E**); *LsPPO7* (**F**); *LsPPO8* (**G**); *LsPPO9* (**H**); *LsPPO10* (**I**); *LsPPO12* (**J**); *LsPPO13* (**K**); *LsPPO14* (**L**); *LsPPO15* (**M**); *LsPPO16* (**N**); *LsPPO17* (**O**). * represents that there are significant differences between different time points and 0 h (*p* < 0.05).

To further investigate the role of *LsPPOs* in lettuce stem browning, the expression levels of 17 *LsPPO* genes at different storage times were measured (Figure 5). *LsPPO5* and *LsPPO11* were not amplified during primer-specific verification PCR and were not expressed in lettuce stems. The expression of most *LsPPOs* fluctuated over time. *LsPPO1*, *LsPPO3*, *LsPPO4*, *LsPPO*8, *LsPPO*9, *LsPPO*10, *LsPPO12*, *LsPPO13*, *LsPPO14*, *LsPPO16,* and *LsPPO17* were upregulated after wounding stress, with peak expression observed at various times: 48/48/72, 24/8/48/48/4, 8, 48, and 24 h, respectively. Among them, *LsPPO4* exhibited the highest upregulation, with a 54.58-fold increase. *LsPPO2*, *LsPPO6*, *LsPPO7*, and *LsPPO15* showed downregulation during storage, reaching their lowest expressions at 4/8/24/48/72 h, respectively. These results suggest that *PPO* played an important role in lettuce stem browning after wounding stress.

### 2.7. Correlation Analysis of PPO Activity and LsPPOs Expression Level with Lettuce Browning

**Figure 6:**
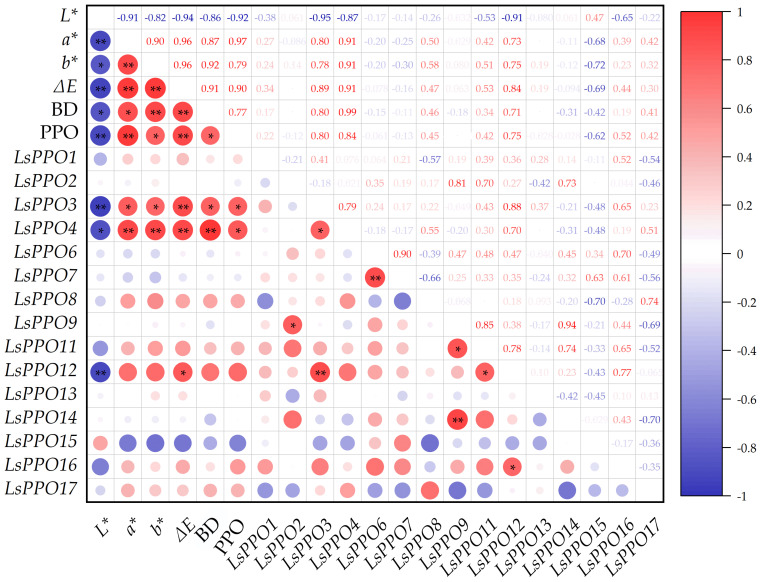
PPO activity and *LsPPOs* expression level were correlated with lettuce stem color change. L*, Luminosity; a*, red/green; b*, yellow/blue; ∆*E*, for differences in color empfindung; PPO, polyphenol oxidase activity. *LsPPO1*-*LsPPO17*, *LsPPOs* gene expression. Red represents positive correlation, blue represents negative correlation; * represents *p* < 0.05); ** represents *p* < 0.01.

A correlation analysis was conducted between the expression levels of *LsPPOs*, PPO activity, and color changes during storage of lettuce stems (Figure 6). The change in PPO activity was found to be significantly negatively correlated with the L* value and significantly positively correlated with a*, ∆*E*, and b* values, as well as with the degree of browning (BD). This indicated that PPO activity was closely associated with browning in lettuce stems. PPO activity showed a significant positive correlation with *LsPPO3*, *LsPPO4*, and *LsPPO12*. Among them, *LsPPO3* and *LsPPO4* were significantly correlated with lettuce stem color changes (L*, a*, b*, ∆*E*, BD), while *LsPPO12* was only significantly correlated with L* and ∆*E*.

## 3. Discussion

PPO is widely distributed in plants, animals, and microorganisms, and is a key enzyme that triggers enzymatic browning during fruit and vegetable processing. With the advancement of molecular biology, *PPOs* from various fruits and vegetables have been cloned and identified. The number of *PPOs* varied across species. For instance, *Salvia miltiorrhiza* had the largest known *PPO* gene family among plants (19 genes), while olive, artichoke, potato and avocado contained 18, 11, 5, and 4 *PPOs,* respectively [25,26,27,28,29]. Interestingly, Arabidopsis lacked *PPO* genes, which was likely due to the independent gene duplication events that shaped its genomic lineage [30]. The number of *PPO* family members did not show a clear correlation with genome size. Based on the available lettuce genome sequences, 17 *LsPPO* members were identified in the study, which was more than that found in artichoke. This suggested that gene duplication events during the evolutionary process contributed to the gene family in lettuce. With the exception of *LsPPO2*, all *LsPPO* genes lacked introns. The absence of introns was also observed in Solanaceae family, including potato and eggplant. In artichoke, each of the four *PPO* members contained one intron, while the rest did not [29]. Studies have shown that introns play a crucial role in transcriptomes regulation, and their absence may accelerate transcription of genes involved in stress or defense responses [31,32]. This suggests that the structure of *LsPPO* genes may support a more rapid response to stress.

Subcellular localization prediction indicated that LsPPO proteins were primarily located in organelles such as chloroplasts and mitochondria, consistent with the subcellular localization of *PPOs* in other species [33]. The hypothesis of phenol enzyme region distribution, which is one of the mainstream theories for enzymatic browning in fruits and vegetables, proposes that phenolic compounds and PPOs are present in distinct cellular regions. Free phenolic compounds are mainly found in vacuoles, while PPOs are concentrated in the cytoplasm and organelle membranes, separated by the plasma membrane [34]. This study further supported the regional distribution of phenol enzymes. PPO proteins on organelles or plasma membranes interacted with phenolic compounds in vacuoles only after cell damage, leading to oxidation.

The phylogenetic tree revealed that artichoke, belonging to the same Asteraceae family, clustered with lettuce in subgroup A. LsPPO1, LsPPO3, LsPPO4, LsPPO5, and LsPPO6 genes in lettuce clustered with CcPPO7 and CcPPO11 in artichoke, while they separated from other LsPPO genes and grouped with PPOs from other species, such as potato and lotus, forming subgroup B. This indicated that certain LsPPO genes were highly conserved, while others underwent functional diversification. *CcPPO7* and *CcPPO11* in artichoke showed significantly higher expression in browning tissues [29], and *StuPPO1* and *StuPOT72* regulated browning in potato tubers [25]. Similarly, *NnPPO1*, *NnPPO* and *Nn-AS-1* also regulated the browning of lotus root [35]. This suggested that the PPO genes in subgroup B, including LsPPO1, LsPPO3, LsPPO4, LsPPO5, and LsPPO6, might play similar roles in browning regulation.

To identify *PPO* genes associated with browning in lettuce stems, RT-qPCR was used to assess the transcription levels of the 15 separated *LsPPO* genes in stem tissues with different browning phenotypes. All *LsPPOs* responded to wounding stress, but the response varied in intensity and timing. *LsPPO1*, *LsPPO3*, *LsPPO4*, *LsPPO8*, *LsPPO13*, and *LsPPO17* were significantly upregulated 4 h after fresh-cut, triggering stress and defense mechanisms, while *LsPPO2*, *LsPPO4*, *LsPPO8*, *LsPPO9*, *LsPPO10*, *LsPPO12*, and *LsPPO14* were upregulated or continuously upregulated after 8 h. Notably, *LsPPO15* was significantly downregulated after fresh-cut and maintained low expression. In litchi, at early stage of postharvest storage, *LcPPO* expression enhanced the PPO activity and accelerated browning [36]. The transcription expression of *PPO3* in avocado also increased browning [26]. Likewise, MdWRKY3 enhanced transcriptional activation by binding to the promoter region of *MdPPO7*, a key gene in the browning of apple [18]. However, in olive, the expression of *OePPO8* did not correlate with browning degree, with the lowest expression observed in the most severely browned fruits. Similarly, the expression of *PPO10* in artichoke was lower in brown callus than that in green callus, suggesting that not all *PPO* genes are involved in browning regulation and might serve other functions. Researches have indicated that *PPO* expression exhibited spatiotemporal specificity and responded to hormones, salt, drought, and low-temperature stress [37,38].

For further verification, we measured PPO activity and quantified color changes in corresponding materials. PPO activity in lettuce increased gradually as browning occurred, which confirmed the crucial role of PPO in the enzymatic browning of fresh-cut lettuce stems, consistent with previous findings [39,40]. Further, correlation analysis of the 15 isolated *LsPPO* genes with their activity and browning degree revealed that *LsPPO3*, *LsPPO4*, and *LsPPO12* were significantly correlated with them. Among these, *LsPPO4* showed the highest and most sustained upregulation, suggesting that it might play a key role in the browning regulation of lettuce.

## 4. Materials and Methods

### 4.1. Plant Materials

Fresh romaine lettuce (*Lactuca sativa* L. *longifolia*) was purchased from the Chengdu market (Chengdu City, Sichuan, China). Twenty lettuce plants with similar sizes (250 ± 50 g), no obvious pests or diseases, and in good health were selected for the experiment. The soil and all leaf tissue were removed from the lettuce, and only the lettuce stems were kept, then rinsed well in distilled water. The lettuce stems were cut into 3–5 cm long pieces, rinsed in distilled water, drained of surface water, and placed in polythene film bags. Each bag contained 100 g of lettuce stem; 21 bags were packed and stored at room temperature (25 °C ± 1 °C, RH 86% ± 3%, dark storage). Samples were taken at 0 h, 4 h, 8 h, 12 h, 24 h, 48 h, and 72 h of storage, and then ground into powder using liquid nitrogen and stored at −80 °C for further analysis.

### 4.2. LsPPOs Gene Family Identification and Physicochemical Analyses

Lettuce genomic data (Lsat_Salinas_v11) were downloaded from Lettuce genome DataBase (https://db.cngb.org/lettuce/ (accessed on 12 August 2024)) to identify the *LsPPOs* gene family. Candidate members of the *LsPPOs* were obtained by searching using HMMER3.2.1 with default parameters (E-value < 0.001) using the Hidden Markov Model files of tyrosinase (PF00264), PPO1_DWL (PF12142), and PPO1_KFDV (PF12143) structural domains as seed sequences. The 11 CcPPO protein sequences from Globe Artichoke were retrieved from the Globe Artichoke Genome Database (https://www.artichokegenome.unito.it (accessed on 14 August 2024)) and used as queries to perform local BLASTP (blast-2.9.0) with an E-value 1 × 10^10^ to obtain the *LsPPOs*. The two results were merged to remove redundant sequences, and the PPO structural domain validation was performed using the NCBI-CDD online tool (https://www.ncbi.nlm.nih.gov/cdd/?term= (accessed on 20 August 2024)) to finally identify the members of *LsPPO* genes family and named them according to their chromosomal location.

Expasy (https://www.expasy.org/ (accessed on 1 September 2024)) was used to predict the molecular weight, isoelectric point, stability coefficient, hydrophilicity and hydrophobicity of PPO protein. The CELLO (CELLO v.2.5) and Wolf Psort online tool (https://wolfpsort.hgc.jp/ (accessed on 1 September 2024)) were used to predict the subcellular localization of members of the *LsPPOs* gene family. The online tool SOPMA (https://www.expasy.org/resources/swiss-model (accessed on 20 September 2024)) was used to predict protein secondary; the conserved motifs of PPO protein were analyzed by MEME (https://meme-suite.org/meme/ (accessed on 1 September 2024)). TBtools v2.119 software was used to visualize gene structure, conserved domains, and conserved motifs.

### 4.3. Codon Usage Characteristics

EMBOSS CUSP (https://www.bioinformatics.nl/emboss-explorer/ (accessed on 1 December 2024)) was used to calculate GC contents at the first (GC1), second (GC2), and third bases (GC3) of codons as well as the average value of GC1 and GC2 (GC). The effective number of codons (ENC) and relative synonymous codon usage (RSCU) were calculated using CodonW1.4.4 software [41].

### 4.4. Phylogenetic Analyses of the PPO Gene Family

The PPO amino acid sequences of rice, potato and lotus root were obtained from the NCBI database [25,29,35]. The phylogenetic tree of lettuce, rice, potato, lotus root and globe artichoke were constructed using MEGAX (version X, Hachioji, Tokyo, Japan) with neighbor-joining method and 1000 bootstrap replicates.

### 4.5. Color

The color of the more uniformly colored parts of the lettuce stems cut surface was measured by a CR-400 colorimeter (Konica Minolta Holdings, Inc., Tokyo, Japan). Three bags were randomly selected at each time point for a total of 20 measured values. The L*, a*, b* and ∆*E* (∆*E* = $\sqrt{{\left(∆L^{*}\right)}^{2}+{\left(∆a^{*}\right)}^{2}+{\left(∆b^{*}\right)}^{2}}$) values were used to evaluate the color change in the lettuce stems.

### 4.6. Browning Degree

The browning degree of lettuce was determined according to the method of Min et al. [42]. An amount of 1 g of lettuce stem sample was homogenized with 5 mL of 0.05 mol/L phosphate buffer (pH 7.0, stored at 4 °C). The homogenates were extracted at 4 °C for 30 min. During the extraction, they were shaken every 5 min, and centrifuged at 4 °C and 8000 rpm for 5 min. The supernatant was taken to determine the absorbance at 410 nm.

### 4.7. PPO Activity

According to the method of Han et al. [43], 1 g sample of lettuce stem was homogenized with 1 mL of 0.1 mol/L phosphate buffer (pH 6.0, stored at 4 °C). The homogenates were centrifuged at 10,000 rpm for 15 min at 4 °C to obtain crude enzyme extract and placed on ice for later use. An amount of 3 mL of 50 mmol/L catechol was mixed with 0.5 mL of crude enzyme extract, and the absorbance at 420 nm was determined immediately. The total determination time was 2 min. The PPO activity increased by 0.1 per minute; absorbance changed per gram of lettuce as an active unit, and the unit was ∆ OD_420_/min · g.

### 4.8. RNA Extraction and Quantitative Real-Time (qRT-PCR) Analysis

Total RNA was extracted using plant RNA extraction kit (Tiangen Biotech Co., Beijing, China), and subsequently converted into first-strand cDNA using the PrimeScript RT reagent Kit with gDNA Eraser (TAKARA, Dalian, China).

A set of primers specific for *LsPPOs* and a pair of primers specific for *LsActin* (Gene ID: XM_023878805.3) were designed using the NCBI-Primer-BLAST (https://www.ncbi.nlm.nih.gov/tools/primer-blast (accessed on 7 November 2024) (Table 2).The qRT-PCR was performed using SYBR Green I kit (TAKARA, Dalian, China) in a Roche LightCycler 96 instrument (Roche, Basel, Switzerland). Relative gene expression was evaluated using the 2^−∆∆CT^ method, with three biological replicates per sample.

## 5. Conclusions

Fresh-cut lettuce is a widely consumed and highly favored vegetable. PPO is the key enzyme responsible for enzymatic browning in lettuce. In this study, 17 *LsPPO* genes were identified from the lettuce genome database and subjected to various bioinformatics analyses, including phylogenetic relationships, gene structure, and domain conservation. Additionally, their expression patterns under wounding stress were investigated. The results indicate a strong correlation between lettuce stem browning and *LsPPOs* expression, with *LsPPO4* potentially playing a critical role in this process.

## Figures and Tables

**Table 1:** Physicochemical properties of *PPOs* in lettuce.

Gene Name	Gene ID	CDS (bp)	Amino Acid	Molecular Weight (KDa)	Average Value of Total Hydrophilicity	Isoelectric Point	Instab-ility Index	Chr	Subcellular Localization
*LsPPO1*	XM_023878429.1	1773	590	66.43	−0.487	5.9	44.69	2	Chlo
*LsPPO2*	XM_042898356.1	1913	618	70.12	−0.584	6.05	41.07	3	Chlo
*LsPPO3*	XM_023907623.3	622	146	15.97	−0.473	4.73	51.66	4	Cyto
*LsPPO4*	XM_023907626.3	1960	596	66.76	−0.547	6.59	45.04	4	Chlo
*LsPPO5*	XM_023907627.3	1964	591	66.47	−0.544	6.29	45.02	4	Mito
*LsPPO6*	XM_023907629.3	1974	603	67.94	−0.595	6.59	49.56	4	Chlo
*LsPPO7*	XM_023909952.3	2018	609	68.52	−0.451	6.27	43.13	7	Pero
*LsPPO8*	XM_023910066.2	1974	615	69.48	−0.501	6.43	43.07	7	Chlo
*LsPPO9*	XM_023910589.3	2030	611	69.8	−0.453	6.04	45.87	7	Chlo
*LsPPO10*	XM_023882917.2	1821	606	69.19	−0.632	5.91	50.39	8	Chlo
*LsPPO11*	XM_023885882.3	2130	612	68.28	−0.38	6	39.43	9	Chlo
*LsPPO12*	XM_023885884.3	2132	634	72.35	−0.534	5.84	35.25	9	Cyto
*LsPPO13*	XM_023885885.3	1232	303	33.74	−0.358	6.86	40.67	9	Chlo
*LsPPO14*	XM_023885921.3	2137	667	75.59	−0.549	6.06	32.42	9	Chlo
*LsPPO15*	XM_023900746.3	2053	610	67.93	−0.334	6.16	34.44	9	Mito
*LsPPO16*	XM_023900747.3	2066	627	71.57	−0.735	8.32	44.73	9	Chlo
*LsPPO17*	XM_023908190.2	2112	591	65.99	−0.514	7.2	45	9	Chlo

Note: CDS, coding sequence length; bp, base pair; KDa, the unit of molecular weight; Chr, chromosome; Chlo, chloroplast; Cyto, cytoplasm; Mito, mitochondrion; Pero, peroxisome.

**Table 2:** Real-time fluorescent quantitative PCR primers.

Genes	Gene ID	Forward Primer (5′–3′)	Reverse Primer (5′–3′)
*LsPPO1*	XM_023878429.1	CTCCTGTATCCTACGACCA	GGAACCAGCACTTTTATCACC
*LsPPO2*	XM_042898356.1	ACTACTGCATGTTGCCCTCC	TTCATGTATCCTTCCGGGGC
*LsPPO3*	XM_023907623.3	GATGAGGATGAGGAGACAGCG	CGTTCCTGTGCTTATGTGGC
*LsPPO4*	XM_023907626.3	ATTGCGATGGGGCATACGAT	CCATCAGGTGCATCCCAGTT
*LsPPO5*	XM_023907627.3	TTCCACCACAGTCAAACCCC	ACCGAGTTGGACATATGCGG
*LsPPO6*	XM_023907629.3	AACAACAAGCAAGCTGTGGC	CCGTGGAACCAGCACTTTGA
*LsPPO7*	XM_023909952.3	CAGCGAAAAACCGAAGCCAA	TCTCGGATGCAGAGCTTGTG
*LsPPO8*	XM_023910066.2	AGGATGATGTCAGGGCAAGC	TAACATGTCGTCACCAGGGC
*LsPPO9*	XM_023910589.3	GGCCTTTACACCACCGTCA	CGCATGTGGAGAAGTTTGGC
*LsPPO10*	XM_023882917.2	CATCCGGAATGTCAATCACCT	GTTGCGCTTCATCTGCCTA
*LsPPO11*	XM_023885882.3	CCAATGACGCTGTAAGGGGT	GAAGGACTGGGTTGGTAGGC
*LsPPO12*	XM_023885884.3	GGAGCTCAGCAGAACGGATT	TTGTTGGTGGTGACTTCGGT
*LsPPO13*	XM_023885885.3	CCTCGGTGGTCTCTTTGTGG	AGCAGCACAAACCGAGTGAT
*LsPPO14*	XM_023885921.3	TTCCCATCGAAGCACCAGAC	CCGATGATGCAAACACCGGC
*LsPPO15*	XM_023900746.3	AGGCCAGCTACAAACAGGAC	ATGTGGCAACTGTGCGAAAC
*LsPPO16*	XM_023900747.3	TGGGCAACTTCTACTCTGCG	TCTGTCGGTTCTTTGCCTCC
*LsPPO17*	XM_023908190.2	ATCTTCAAGTGGATTTCCCGAT	CTCCCCAGGATTCTCTCGT
*LsActin*	XM_023878805.3	AGCAACTGGGATGACATGGA	GGGTTGAGAGGTGCCTCAGT

## Data Availability

Data are contained within the article.

## Supplementary Materials

The following supporting information can be downloaded at: https://www.mdpi.com/article/10.3390/plants14060972/s1, Figure S1. Roman lettuce for testing; Table S1. Prediction of PPO family protein secondary structure in lettuce; Table S2. Correlation parameter of codon usage bias in *LsPPOs*; Table S3. Statistics of codon bias of *LsPPO* family genes.
