# Supplementary material for: Identification and Expression Analysis of Polyphenol Oxidase Gene Family Members in Response to Wound Stress in Lettuce (Lactuca sativa L.)
Source: Plants (Basel). 2025 Mar 19;14(6):972. doi: 10.3390/plants14060972 (PMC11945535; doi:10.3390/plants14060972)
Supplement: Supplementary file 1 [file plants-14-00972-s001.zip › plants-3517756-supplementary.pdf]

Figure. S1 Roman lettuce for testing.

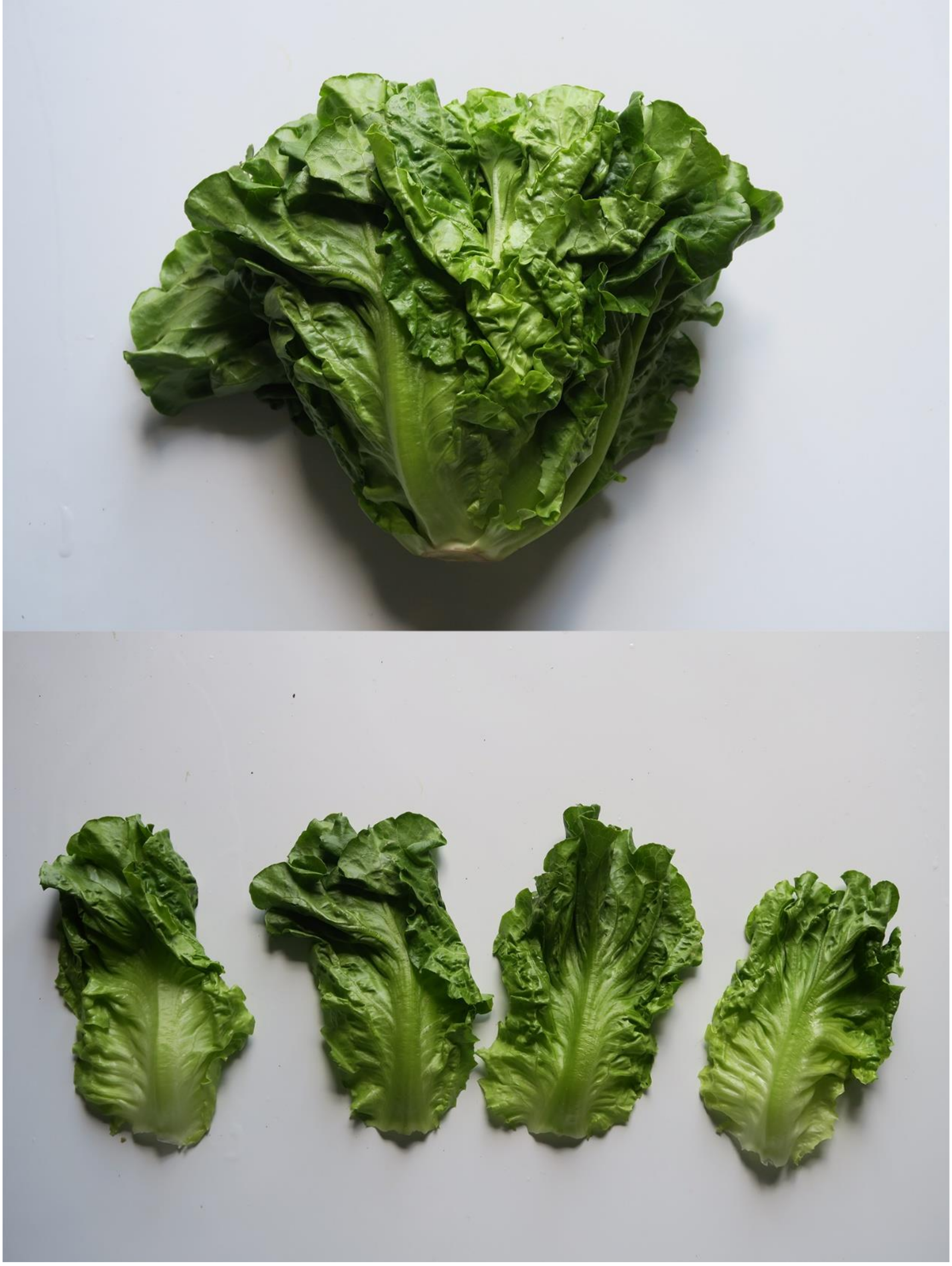

Table S1. Prediction of PPO family protein secondary structure in lettuce.

Gene	Alpha helix / %	Beta turn / %	Extended strand / %	Random coil / %
LsPPO1	15.93	0.34	12.2	71.53
LsPPO2	15.86	0.32	12.62	71.2
LsPPO3	19.86	2.74	23.97	53.42
LsPPO4	14.93	0.5	11.58	72.99

LsPPO5	15.57	0.51	11.34	72.59
LsPPO6	15.42	0.66	12.11	71.81
LsPPO7	15.76	0.66	11.66	71.92
LsPPO8	15.61	0.33	11.54	72.52
LsPPO9	16.2	0.82	13.26	69.72
LsPPO10	15.18	0.99	12.87	70.96
LsPPO11	15.03	0.65	13.73	70.59
LsPPO12	16.03	0.48	11.38	72.12
LsPPO13	19.47	0.99	23.76	55.78
LsPPO14	18.29	0.75	13.04	67.92
LsPPO15	17.38	0.66	13.93	68.03
LsPPO16	15.47	0.32	11.96	72.25
LsPPO17	15.06	0.85	12.86	71.24

Table S2. Correlation parameter of codon usage bias in *LsPPOs*.

Gene	GC				ENG
	GC1	GC2	GC3	Total GC	
LsPPO1	48.56%	39.76%	39.09%	42.47%	53.64
LsPPO2	48.79%	40.55%	49.92%	46.42%	58.57
LsPPO3	30.92%	39.13%	48.79%	39.61%	49.76
LsPPO4	51.93%	40.54%	44.72%	45.73%	57.44
LsPPO5	51.18%	39.19%	41.55%	43.98%	54.54
LsPPO6	50.17%	39.74%	44.04%	44.65%	55.53
LsPPO7	52.46%	38.69%	49.51%	46.89%	58.99
LsPPO8	50.00%	39.45%	49.35%	46.27%	55.22
LsPPO9	49.02%	39.05%	51.14%	46.41%	56.84
LsPPO10	49.42%	39.70%	49.26%	46.13%	60.31
LsPPO11	50.57%	39.15%	54.65%	48.12%	56.31
LsPPO12	49.61%	39.06%	50.87%	46.51%	57.87
LsPPO13	48.68%	36.18%	50.66%	45.18%	56.81
LsPPO14	50.60%	38.77%	49.55%	46.31%	61
LsPPO15	51.55%	39.93%	49.59%	47.03%	58.13
LsPPO16	49.20%	40.13%	49.52%	46.28%	58.24
LsPPO17	50.84%	41.55%	55.91%	49.44%	55.06
Average Value	49.03%	39.45%	48.71%	45.73%	56.72

Note: GC1, GC2, and GC3, respectively, represent the GC content of the first, second, and third bits of the codon; total GC represents the total GC content of the codon; ENC indicates the number of effective codons.

Table S3. Statistics of codon bias of *Lactuca sativa* L. PPO family genes.

Amino Acid	Codon	Number	Relative Synonymous Codon Usage	Amino Acid	Codon	Number	Relative Synonymous Codon Usage
TER*	UAA	11	0.9706	Met	AUG	217	3
	UAG	4	0.3529		CUG	0	0
	UGA	19	1.6765		UUG	0	0
Ala	GCA	165	1.0217	Asn	AAC	303	1.0669
	GCC	160	0.9907		AAU	265	0.9331
	GCG	94	0.582	Pro	CCA	227	1.4367
	GCU	227	1.4056		CCC	115	0.7278
Cys	UGC	64	0.805		CCG	113	0.7152

	UGU	95	1.195		CCU	177	1.1203
Asp	GAC	313	0.8817	Gln	CAA	169	1.295
	GAU	397	1.1183		CAG	92	0.705
Glu	GAA	265	1.0173	Arg	AGA	126	1.5949
	GAG	256	0.9827		AGG	126	1.5949
Phe	UUC	262	1.1055		CGA	60	0.7595
	UUU	212	0.8945		CGC	50	0.6329
Gly	GGA	133	1		CGG	48	0.6076
	GGC	108	0.812		CGU	64	0.8101
	GGG	112	0.8421	Ser	AGC	103	0.9129
	GGU	179	1.3459		AGU	114	1.0103
His	CAC	114	0.8352		UCA	145	1.2851
	CAU	159	1.1648		UCC	127	1.1256
Ile	AUA	112	0.7015		UCG	53	0.4697
	AUC	183	1.1461	Thr	ACA	144	1.0492
	AUU	184	1.1524		ACC	183	1.3333
Lys	AAA	351	1.0308		ACG	78	0.5683
	AAG	330	0.9692		ACU	144	1.0492
Leu	CUA	57	0.4783	Val	GUA	82	0.5458
	CUC	150	1.2587		GUC	145	0.9651
	CUG	75	0.6294		GUG	199	1.3245
	CUU	153	1.2839		GUU	175	1.1647
	UUA	92	0.772	Trp	UGG	151	1
	UUG	188	1.5776	Tyr	UAC	210	1.1932
Ser	UCU	135	1.1965		UAU	142	0.8068

Note: * represents the stop codon. Ala: alanine, Cys: cystine, Asp: asparticacid, Glu: glutamicacid, Phe: phenylalanine, Gly: glycine, His: histidine, Ile: isoleucine, Lys: lysine, Leu: leucine, Ser: serine, Met: methionine, Asn: asparagine, Pro: proline, Gln: glutarnine, Arg: arginine, Thr: threonine, Val: valine, Trp: tryptophan, Tyr: tyrosine.
